# The relationship between perceived institutional conditions and firm-level innovations in emerging markets: Moderating effects of firm ownerships

**DOI:** 10.1371/journal.pone.0291290

**Published:** 2024-01-22

**Authors:** Samuel Amponsah Odei

**Affiliations:** Department of Economics, University of Hradec Králové, Hradec Králové, Czech Republic; University of Almeria, SPAIN

## Abstract

Country-level institutional conditions are known to play key roles in firms’ innovation and operations. While it is acknowledged that an unfavourable institutional context negatively influences innovation, less is known about which firms are most affected by these institutional conditions. This research aims to examine which institutional conditions affect firms’ product innovation and which firms can innovate their products despite the unfavourable institutional environment. The empirical results based on 21,056 firms from 42 African countries show that legal institutions and informal competition positively influence product innovation while perceived policy instability has a negative influence. The results proved that the interactions between perceived policy instability and favourable legal institutions negatively influence product innovation. However, the joint effects of these institutional conditions positively impact product innovation. Private domestic ownership positively moderates the relationship between a favourable legal environment and product innovations, while foreign ownership negatively moderates the relationship between a favourable legal environment and product innovations. Finally, the results showed that both domestic and foreign ownerships negatively moderate the relationship between perceived policy instability and product innovation. The main practical implication from our result is that policy practitioners in African countries should endeavour to reduce the potential negative impact of the policy instability and unfavourable legal environment for foreign-owned firms as it reduces their incentives to innovate.

## Introduction

The rapid rate of globalisation has led to enlarged markets and offered several advantages to firms such as increased revenues and productivity [1, 2]. At the same time, it exposes businesses to intense rivalry from other foreign firms, provides network opportunities, and access to new technologies and knowledge, among others. The adoption of innovation has been heralded as a remedy for firms to overcome competitive pressure and other organisational challenges. Innovation allows firms to significantly improve their products and services, marketing, and organisational activities, leading to improved productivity and competitiveness. Despite its growing importance, there is a considerable divide in the intensities of innovation between the developed and emerging economies. Firms in developing economies face several challenges, such as low public support for innovation and inadequate access to innovation funding, as well as other institutional challenges that negatively impact the ability to successfully innovate. Developing countries are characterised as having weak institutions that negatively influence the success rate of innovations. Institutional factors such as political uncertainty, fiscal policies, legal bodies, corruption, regulatory quality, and informal competition limit enterprises’ abilities to innovate in developing and transition countries [3]. In these countries, the business environment is defined by embedded economic institutions, namely regulatory quality, rule of law, corruption control as well as government effectiveness.

Several strands of research have indicated that perceived institutional conditions affect firms’ operations, and this is widespread in emerging and developing countries [3, 4]. In countries with stable and well-functioning institutions, the potential risks related to introducing innovation can be diminished [5]. These well-functioning institutional conditions could help shape firms’ internal capabilities, which is a basic precondition for innovation to thrive and succeed [6, 7]. An inimical institutional environment could serve as an impediment to firm-level innovations due to its ability to increase the expenditure involved. Rodríguez-Pose and Zhang [8], posit that weak institutional conditions serve as considerable impediment to innovation, whereas better institutions reduce firms’ time spent dealing with excessive government regulations. Perceived policy instability could for instance, strengthen firms’ excessive reliance on governments, paving the way for them to engage in non-market policies, specifically engaging in corrupt practices, to avoid the government’s transaction procedures. Economic institutional conditions, like corruption, weak rule of law, as well as regulatory quality, could also deteriorate firms’ innovation investments. Effective competition rules administered by an efficient and well-organised legal system could enhance innovation. Efficient legal systems could also stimulate innovations by strengthening contract enforceability [9] and ensuring that firms do not spend innovative times in court rooms. Similarly, informal firms’ competitive pressure can also pose a problem for innovative firms’ operations. These competitions from informal firms affect the innovation strategies of formal innovative firms, hence serve as a disincentive to introducing new products or services [10].

In African countries, research on firm innovations is on the rise (see, for example, [11]). However, these flourishing research displays several limitations that still limit the complete understanding of the innovation landscape in these countries. Research that has analysed the relationship between institutional conditions and how they affect firms’ innovations is limited. Several of these studies have focused on political instability and its influence on innovations (see, [12, 13]) and corruption [14]. However, other perceived aspects of institutional conditions, like informal sector competition and legal institutions, are yet to receive ample scholarly attention. Another aspect where existing research shows caveats is their neglect of the interactions between institutional conditions embedded in the businesses environment and how they jointly affect firms’ abilities to innovate. We argue that firms are influenced by various institutional conditions simultaneously and their joint impacts need to be examined in relation to their abilities to influence firms’ innovations. We argue that the neglect of these perceived factors do not make our understanding of the nexus between perceived institutional conditions and innovations complete, as they can negatively influence firms’ innovations. Furthermore, while we know that an unfavourable institutional context negatively influences innovation, no study in African countries has focused on investigating which firms are most affected or not by perceived institutional conditions. This therefore calls for new research to examine which firms can innovate their products and processes despite the unfavourable institutional environment. The non-inclusion of these critical aspects of institutional conditions reduces our understanding of the innovation ecosystem in African economies and therefore calls for new research that integrates all these ignored but important factors to provide a far-reaching understanding of firm-level innovation. This research fills these identified research gaps by exploring the combined impacts of legal systems, perceived policy instability, and informal sector competitions’ role in influencing firm-level innovations. Unlike previous studies, this study measures these institutional conditions with whether managers perceive that a particular institutional factor is an obstacle to their operations. The main advantage of this approach is that it helps to provide subjective and valuable insights into managerial experiences and opinions on these institutional conditions [15]. We also argue that perceptions could provide detailed contextual understanding of the nuances and complexities of these institutional conditions, as they consider the socio-cultural and historical factors that influence managers views and opinions. This article focuses on firms in African countries characterised as having weak institutions and aims to investigate the relationship between these perceived institutional conditions (and their combinations) and firms’ innovation. We also explore whether ownership structures moderate the relationships between institutional conditions and firms’ innovations. We provide theoretical contributions and a convincing path for the improvement of African firms’ innovation by providing policy implications that could be applicable to other developing economies.

This article is organised in the following manner: Section one introduces the paper with a general overview of the association between perceived institutional conditions and firms’ innovations, the research gap, and novelty contributions; section two is devoted to reviews and discussion of existing literature on the concepts of perceived institutional conditions and how they shape innovations in African countries. Section three centres on the data source, research method, and description of variables used for the empirical model specification; section four is dedicated to the detailed result discussions relating to existing studies. The last section ends the study with recommendations for future research direction, practical and policy implications, and research limitations.

### Theoretical background and hypotheses formulation

The implementation of innovation has become one of the most prominent strategic assets firms can utilise to advance their performance, competitiveness, and overall productivity. The continued developments in firms’ innovation proficiencies are beneficial to improving and maintaining competitiveness in the fast-paced and dynamic market environment. Innovation in new product development is crucial for firms to achieve and maintain their competitive advantage [16]. Product innovation designates firms’ propensity to offer significantly better-quality products or processes to the market earlier than competitors [17]. Product innovations entail firms putting in concerted efforts to significantly improve upon their existing products, processes, and services such that they become considerably new to the firm. These offered goods and services should be new or have considerably improved qualities to enhance customer satisfaction. Product innovation thus incorporates both new product and process development in addition to new functional uses for existing products. Implementing product innovations requires firms to undertake a wide array of activities either independently or in close partnership with other partners that should significantly lead to advancements in product and service quality and provide economic gains to firms. To successfully innovate new products and services, firms need to embark on several industrial undertakings, such as engaging in research and development, making advanced modifications to existing production processes, investing in new equipment, human capital, and changing management practises [18–20].

The theoretical foundation of this article is the institution-based view, which posits that there exist dynamic relationships between institutions and organizations and that firms’ strategic choices are based on such interactions [21]. These interactions mean that firms actively react to both existing formal and informal institutional conditions [3, 22]. Several attempts have been made by different scholars aimed at providing definitions of what institutions entail [23]. In the view of North [24], institutions could be explained as "the humanly devised constraints that structure human interaction" (p.97). Similarly, Greif and Laitin [25] provide an alternative definition of institutions, which they define as "a system of human-made, nonphysical elements—norms, beliefs, organizations, and rules—exogenous to each individual whose behaviour it influences and that generates behavioural regularities" (p. 635). Institutions can therefore be understood as the humanly developed structures consisting of norms and rules that affect and restrict human and organisational behaviour. The interactions between organisations and institutions provide these organisations with opportunities to advance coping strategies to overcome the negative effects of institutional actions [3]. Institutions dictate the "rules of the game" as they guide firms to make well-informed strategic choices [21]. According to North [24], institutions can be broadly grouped as formal and informal, where formal institutions involve a set of rules designed to regulate human behaviour. They are developed, conveyed, and discharged using official organisations such as law courts, parliaments, and other bureaucracies. Sinani et al. [26] describes formal institutions as a multidimensional concept comprising economic, legislative, and political systems. Contrary to this, Pejovich [27] defines informal institutions to comprise of the "traditions, customs, moral values, religious beliefs, and all other norms of behaviour that have passed the test of time" (p. 166).

These external institutional factors or conditions could affect firms’ operational activities as they are embedded in the business environment. These institutional effects could shape firms’ behaviour and strategies. Governments have been entrusted with the powers to ratify and enforce rules and laws, and these responsibilities could impact firms’ operations and growth opportunities [3, 28]. Firms and individuals are obliged to follow the various rules and laws made by governments, and this shapes most business aspects. Government rules also help determine whether firms can operate ethically and legally or not. Prevailing institutional conditions established in a specified geographic location will not only impact firm activities but could also impede them, necessitating research to establish the impact of such conditions [29]. However, institutional effects may be perceived differently by firms, probably since they may have different connections with the government or its branches, such as the judiciary. Firms may also perceive these institutional effects differently because they may have different levels of experience confronting them because of their managerial experience. Finally, they may be less affected by these external institutional factors, probably due to their size and age. These institutional conditions can therefore act as barriers that could negatively affect firms’ operations, including innovation activities [30]. Several institutional conditions embedded in the business environment, namely policy instability, the legal environment, competition from informal firms, labour regulations, corruption, and tax administration, could negatively impact firms’ abilities to innovate successfully [3, 7]. The combination of these institutional conditions in a business environment could act as hindrances to firms’ operations and entrepreneurial activities.

Stability in the political environment could be vital in enhancing firms and individual behaviour and this has been proven to be a significant risk factor in most developing countries [12]. The existence of unstable and consistent government surges market friction as well as cost and ease of doing business making it challenging to determine business conditions [31]. Political instability results in frequent changes in government as well as governmental policies. Governments are known to play key roles in transition and developing economies especially when it comes to allocating scarce public resource. In transition and developing economies where institutions are known to be unorganized, it places so many responsibilities on the state to fulfil this resource allocation function. Frequent government changes create policy instability which in turns encourages uncertainties and insecurities, which culminate and serve as strong barrier to sustainable innovation [13, 32]. Political instability is associated with inefficient institutional development, low levels of innovation, slow economic development, and low investment inflows [33]. Policy instability could discourage firm-level innovation investments because it could result in firms abandoning such investments when they feel that changes in government could result in policy changes. Firms operating in countries with unstable political environment face significant challenges that can subsequently affect their innovation performance. These challenges from an unstable political environment could serve as a hindrance to attracting foreign direct investment which has been proven to be avenues for firms to absorb new knowledge and foreign technologies knowledge [34]. Policy instability could also impede firms’ innovations by limiting their access to financial resources a vital element for innovations to thrive [35]. Volatile political situations could make financial institutions more reluctant to provide loans owing to the potential high risk of non-payments [36]. Research by Krammer and Kafouros [13] study in sub-Saharan African countries revealed that political instability is negatively correlated with product innovations. Unlike Krammer and Kafouros [13] study, we consider the instabilities in policies these frequent changes in government brings and access whether these perceptions of policy changes affect firms’ abilities to innovate. Based on this understanding, we distinguish between perceived and objective policy instability. Perceived policy instability denotes the subjective opinion or beliefs of individuals or businesses related to the stability or predictability of government policies. They are usually based on the personal interpretations and understanding attached to policy actions and may not necessarily be parallel with the actual magnitude or frequency of the intended policy changes. Contrary, objective policy instability refers to the specifiable and measurable frequency of policy changes, it specifically spotlights the real incidence and characteristics of policy changes, their amendments as well as reversals. Objective policy instability can be measured through various indicators, such as the number of policy changes [37], legislative turnover [38], or the duration of policy implementation [39]. Since the Enterprise Survey is based on respondents’ opinions, we conjecture that perceived specific country-level volatile political conditions could result in policy uncertainties. This can negatively affect firms’ abilities to innovate by impacting resources availability as well as the enticement structures pertaining to firms’ innovation decision.

Innovative firms are considerably influenced by the activities of informal sector firms, and this has been established by the literature (see, for instance, [40]). Informal competition refers to the unwholesome competitive practises of informal firms that can restrict new product and process innovation development of innovative formal sector businesses [41]. In emerging and transition countries, informal firms are predominant, sometimes accounting for more than 80% of all enterprises [42]. Although they are known to play key roles in the economic health of countries, their activities are known to affect the operations of formal sector firms. They usually engage in abnormal competitive practises that can inhibit formal-sector firms’ innovation activities. Informal sector firms engage in activities such as evading taxes and regulatory non-compliance; these behaviours permit them to have relative cost advantages over formal sector firms [43]. Informal sector firms are characterised as being unproductive and inefficient, overly reliant on labour as a production factor, and having inadequate access to financial resources [44]. Two opposing positions have been advanced related to the nexus between informal competitions and how it influences firms’ innovations. The first refers to this correlation as the inverted-U shape or the "Schumpeterian effect" (see, for instance, [45]). The Schumpeterian proponent considers vertical innovation as the "creative destruction" of product market competitions, resulting in long-term growth. Competitions from informal firms therefore serve as a barrier to innovations because they reduce the enticements to carry out innovations and their related set of activities [45]. The second school of thought believes there exists a positive correlation between innovation and informal competitions, or what has become known as the "escape-competition effect" (see, [46]). Research by Pérez et al. [46] in the People’ Republic of China revealed that competition from informal firms has a beneficial influence on formal businesses’ product innovation rates. In agreement with the above-mentioned study, we anticipate that informal competitions could influence formal firms’ resource allocation decisions. When formal firms envisage competitive pressure from the informal sector, they may respond by allocating more resources towards engaging in R&D and innovation to differentiate themselves from their competitors. They may invest in new technology acquisitions, hiring skilled individuals, or engage in collaboration with external partners to boost their innovative capabilities. Consequently, informal competition is expected to positively influence new product development. This study, however, differs from Pérez et al. [46] study in the sense that although their measure captured managerial perceptions, they narrowly focused on China which may not provide a true reflection of the state of informal competition influence on firm-level innovation due to the limited variability in their sample.

The linkage between the legal systems’ influences on firms’ innovation is gaining scholarly attention in recent times (see, for instance, [40]). The judicial system could simultaneously be a facilitator as well as a hindrance to firms’ innovation outcomes. The judicial system sets the limits that define how economic activities must take place and serves as an institution that promotes business activities, including innovations. From the facilitating point of view, the government and judicial systems influence property and contact regulations, which affect existing working conditions. The market system requires strict property and contract laws to thrive, and they can also influence innovations. Labour laws in a country can have a significant impact on a country’s level of innovation [47]. The legal system enforces the rule of law that guarantees swift arbitration between economic agents by extending legal rights associated with intellectual property protection, innovation, and knowledge management [48]. The legal system also safeguards the rigorous enforcement as well as the conformity of existing intellectual property rights laws, which is fundamental to improving the innovation environment [49]. The legal or judicial system exists to also protect economic agents (firms and individuals) from the potential externalities of innovation. The judicial system constantly provides businesses and individuals with inducements to invest in innovations as well as in R&D, which is proven to be a facilitator for innovation development. The law also facilitates social and innovation collaboration, which is vital for innovation promotion and sustenance. Several studies (see, for instance, Usman et al. [50] in 41 developing countries) found that a well-organised legal environment accelerates firm-level innovations. Due to the weak institutional environment in African countries, we expect that an effective and supportive legal framework could encourage and safeguard innovation, as it could provide firms with the certainty and incentives to invest in R&D. As described above, this study considers whether firms perceived the legal system as an obstacle to their operations and was not based on objective measures. Based on the foregoing discussions, we provide our first three hypotheses as follows:

Hypothesis 1a: Perceived unfavourable informal competition is positively correlated with product innovations.
Hypothesis 1b: A perceived favourable legal system is positively related to firms’ product innovations.
Hypothesis 1c: Perceived policy instability is negatively interrelated with product innovations.

### The interactions between perceived policy instability, informal competition, and legal system

The combination of perceived policy instability, informal competition, and legal system can significantly have implication for firms’ innovation outcomes and the overall business environment [3]. Stable and predictable policies, a robust legal system and fair competition are all essential in creating a favourable environment conducive for innovations to thrive. The joint existence of these three institutional conditions in a business environment could impede firms’ abilities to innovate their new products and services. Their combinations create uncertainties, increase risk, and undermine firms’ incentives to invest in and sustain new product development. For instance, unstable policies, coupled with weak judicial system as well as unfair competition from informal sector firms could plunge firms’ incentives to innovate their products [40]. These conditions raise uncertainties rendering the business environment unstable, hence could compel firms to divert scare resources intended for innovations into short-term goals which could help survive the negative impacts of these institutional conditions. In an environment with levels of perceived policy uncertainties and intense informal competition could make firms more risk-averse because they be afraid to invest in innovation activities. They may therefore shift their attention to short-term survival strategies to withstand the competitive pressure rather than investing in research activities. Furthermore, perceived policy instability and a weak legal system could create hurdles in attracting investments as well as attracting and securing innovation funding [51]. This is because investors prefer stable and reliable legal systems to safeguard these investments. Therefore, uncertainties caused by unstable policies and a weak legal environment can deter potential innovation investors leading to a situation where there will be inadequate innovation funding. This situation to diminish firms’ incentives to invest in new product development. The interaction between informal competition and a stronger legal system could also be anticipated to create a dynamic and supportive environment for new product development. Informal competition could create a sense of urgency for firms to have the competitive edge to differentiate themselves from their competitors [46]. When combined with an effective legal system that ensures intellectual property protection, firms are incentivised to invest in innovation to produce novel products and services. Patents and other intellectual property rights granted by the legal system allow innovators to enjoy brief monopolies, recoup their investments, and reap the benefits of successful innovation. We therefore expect that when firms perceive the legal system to be effective, it could have a positive moderating influence, allowing formal firms to continuously engage in innovation to have a competitive advantage.

The joint existence of these three institutional conditions in a business environment could also be expected to positively impact product innovation. When there is high perceived policy instability amidst an effective legal system, it could create a favourable environment for innovation to thrive. This is because a strong and effective legal system can counterbalance the deleterious impacts of policy instability on product innovation. An effective legal environment provides a greater guarantee that innovations will be safeguarded, even amidst perceived policy changes [40]. As a result, firms may be more eager to face off against informal competition because they have the assurance of their ability to protect their intellectual property rights through the legal system [52]. Generally, an effective legal system can act as a buffer against policy instability, enabling firms to navigate uncertain policy environments with assurance. When informal competition is present amidst an uncertain policy environment, the legal system’s effectiveness can positively influence innovation by encouraging firms to embrace informal competition. The favourable legal environment will enhance investments in R&D and ensure intellectual property protection, even in the face of policy uncertainties. Based on the aforementioned discussions, we hence provide our second set of hypotheses.

Hypothesis 2a: The interaction between perceived policy instability and informal competition is expected to negatively influence product innovation.
Hypothesis 2b: The combination of perceived policy instability and fair legal system is expected to negatively influence product innovation.
Hypothesis 2c: The interaction between perceived fair legal system and informal competition is anticipated to positively influence product innovation.
Hypothesis 2d: The moderating effect of informal competition on the relationship between perceived policy instability and product innovation improves when the legal system is effective.

### The moderating role of ownership structures

The ownership structures of firms could play vital roles in influencing product innovation outcomes [53]. Firm owners can influence innovation performance by engaging in new initiatives targeted at influencing their aptitudes to introduce innovative products and processes. Domestic-owned firms could be less probable to be impacted by unfavourable institutional conditions. Domestic firms are usually more acquainted with the prevailing local institutional environment, involving regulations, political dynamics, and cultural norms. They are more likely to developed coping strategies and operational practices that help them to align or adapt to these unfavourable institutional conditions over time. Their experience and understanding of the local context can help them navigate and mitigate the negative effects of unfavourable institutional conditions [54]. Domestic firms tend to have stronger networks and relationships within the local business ecosystem. These relationships can provide them with access to resources, information, and support that can help them overcome institutional challenges [55]. Established connections with government entities, industry associations, suppliers, and customers can provide a level of insulation against unfavourable conditions. Foreign ownership corresponds with an increased possibility of innovations because of their continuous spending on innovation and R&D activities [56]. According to this research, foreign-owned businesses are more probable when compared to domestic firms to introduce innovative products or processes. Foreign-owned businesses also profit from both economies of scale and scope; they are well-endowed with strong financial capacity to spend on innovation. We therefore anticipate that despite foreign firms’ likelihood of being innovative, their innovation performance may be constrained by unfavourable perceived institutional conditions. They may lack experience working in unfavourable institutional settings. They may not have a fair amount of knowledge on how to overcome such perceived conditions, and this can negatively affect their abilities to innovate successfully. For example, D’Souza and Kaufmann [57] discovered that foreign-owned businesses are less probable to engage in bribery compared to domestic owned firms. This behaviour could affect how they deal with perceived institutional conditions such as informal product market competitions and legal institutions. Therefore, we anticipate that foreign ownership will negatively moderate the relationship between institutional conditions and product innovation. Contrariwise, state-owned enterprises’ (SOEs) operations are guided by law, and they are state-supported or funded. Corporate governance in SOEs implies that governments are highly engaged in every decision because of their legal structure, with the government being the principal shareholder [58]. SOEs direct affiliation with the state means that they will be immune from perceived institutional conditions such as dealing with the legal system and informal product market competitions, among others [59]. This immunity they benefit from the state means that they will not be negatively impacted by perceived institutional conditions, and they are more likely to innovate amidst these unfavourable perceived conditions. We therefore summarise the understanding that domestic firms will be able to innovate amidst unfavourable institutional conditions because they know and understand the local prevailing conditions, but foreign-owned businesses are not expected to innovate in such unfavourable perceived conditions. SOEs will also be able to successfully innovate due to the continuous state support, and the state is expected to shield them from perceived negative institutional conditions. Hence, SOEs are not expected to be impacted by the expected negative impacts of institutional conditions. Based on the above-mentioned arguments we provide our remaining hypotheses on the moderation role of various ownership structures on product innovation:

H3: Domestic ownership positively moderates the relationships between perceived institutional conditions and product innovations
H4: Foreign ownership negatively moderates the relationship between perceived institutional conditions and new product development
H5: State ownership will not moderate the relationships between perceived unfavourable institutional conditions and product innovations.

The innovation literature has well determined the nexus between top managers’ years of experience and its probable influence on firm-level innovations (see, [60]). Top managers with more diverse years of service benefit from previous experiences, which can improve the quality of the managerial teams’ abilities to influence innovations (see, [60]). Experienced top managers with more years of service benefit from previous experiences, which can improve the quality of the managerial team. They can capitalise on the improved managerial quality to influence their present innovative performances [61]. Managers with enough experience are anticipated to have familiarised themselves with previous unfavourable institutional conditions such as policy instabilities unfavourable informal competitive pressures, among others, and devise ways to overcome their negative influences on innovation. Empirical studies have proven that experienced managers understand the dangers posed by the informal competitive environment better, and this has a positive influence on their innovative performances [62]. When compared with new managers without more years of experience, they may also learn from the perceived institutional conditions embedded in the market environment, so they are likely to innovate amidst such negative conditions. New managers without many years of service may not have experience dealing with such unfavourable institutional conditions, so they will resort to experimenting with their policies and decisions. They are likely to use the trial-and-error approach because of their lack of previous experience dealing with these perceived institutional conditions. They will need enough time to learn and apply new approaches, which can slow the innovation process [63]. As a result, we anticipate that managerial experience will be critical in firms’ ability to innovate in the face of perceived unfavourable institutional conditions.

The size of firms could also play key roles in firms’ propensity to innovate amidst perceived institutional conditions [3]. By size, firms could be classified as either small, medium, or large. Due to their diverse qualities, resources, and organisational capacities, small and large-scale enterprises frequently demonstrate different propensities to innovate [64] in the face of perceived institutional conditions such as policy instability, the legal environment, and informal competition. Policy instability could exert a more significant impact on small-scale firms because they typically have limited resources and competence to adapt swiftly to changing regulations [65]. Hence, small firms may perceive policy instability as a higher risk, leading to cautiousness in investing in innovation and its related activities. Contrariwise, large-scale firms frequently have more resources, established systems, and access to experts to effectively address policy changes [16]. Large firms may also use their influence and lobbying ability to modify policies in their favour or reduce negative consequences. In comparison to small firms, large firms may have more resources, experience, and capacity to navigate the legal system efficiently. Their resource abundance could mean that they can engage in legal actions to shield their intellectual property and technologies. Formal small firms may struggle to compete with informal firms, whether small or large, because of their resource constraints. This could mean that even though they could envisage competitive pressure from the informal sector, they may not have the resources to have a competitive edge. They may engage in niche strategies, by concentrating on narrow market segments in which they can differentiate themselves through innovation [66]. Contrary, large firms have the resources to engage in radical innovation to gain large market shares and have the competitive advantage over their market rivals. Therefore, we summarise the understanding that small firms may face more significant challenges in adapting and innovating amidst these institutional conditions due to their limited resource endowment, while large firms can benefit from their scale and capabilities to navigate these factors more effectively.

## Data and methodology

The empirical specification and hypothesis testing data are based on a sample of firm-level data from the World Bank’s Enterprise Survey (WBES). The Enterprise Survey is jointly managed by three main financial institutions, namely the World Bank Group (WBG), the European Bank for Reconstruction and Development (EBRD), and the European Investment Bank (EIB). Currently, WBES is carried out in about 154 countries, providing an expansive range of economic data on more than 180,000 businesses. The WBES collects data by means of the stratified random sampling method based upon firm size, industry, as well as geographic region. The WBES has a broad array of business environment data covering topics such as innovations, innovation collaborations, infrastructure, access to funding, corruption, competition, firm characteristics, and performance measures, among others. The WBES is administered to firm owners as well as top managers in the private sector. The comprehensive nature of this survey makes it one of the best datasets for firm-level innovations and institutional condition analysis. Usage of the WBES dataset has soared among researchers, and it has been widely used for several institutional conditions and firm-level innovation analyses (see, [40]). In total, 34,598 firms from 42 African countries were included in the observed sample. However, after cleaning the data and getting rid of missing data and don’t know spontaneous responses, the final sample was reduced to 21,056. The sample included pooled cross-sectional data involving all rounds of survey data for the selected countries over different time periods. The pooled cross-sectional nature of the data means that some firms appear multiple times in different rounds (periods) of the survey, but this was just in few selected countries.

For the methodological approach, we employed the probit regression model because our outcome variable is dichotomous. Therefore, the binary dependent regression model was used to estimate the likelihood that the outcome variable, *y* = 1 is a function of the vector of the covariates *x*. Thus

$$P_{r}\left[y=1Ix\right]=\phi \left(x^{t}\beta \right)$$ (1)

Where,

*ϕ* refers to the aggregate distribution function based on the standard normal distribution; *β* denotes the calculated parameters. We provide our expected model as.

$$y=x^{t}\beta +\varepsilon$$ (2)

Where

*y* is represented by firms’ product innovations. The perceived institutional condition variables thus perceived policy instability, informal competitions and legal system, and other control variables–firm size and age, managerial experience, and various ownerships are denoted by *x*. The error term *ε* is expected to be normally distributed having a mean value of 0 and standard deviation of 1.

## Measurements

### Dependent variable

In agreement with the literature, see Odei and Appiah [2], our outcome variable product innovation measures whether firms have introduced any new or significantly improved products or processes in the last three years. Following the literature, we used a proxy for product innovation; a dummy variable with the value of 1 if firms reported introducing a new or significantly improved product or service, and 0 if otherwise.

### Independent variables

*Informal competition*: refers to the unwholesome competitive practises of informal firms that could hamper the activities of formal innovative firms. Informal competition is a measure of product market competition. Following the recent literature (see [67]), we used a binary variable that signifies whether firms identify informal competition as an obstacle to their operations, with 1 meaning yes and 0 meaning no.

*Perceived policy instability*: In the WBES, firms are asked whether they perceive political instability as an impediment to their functions. Policy instability indicates the extent to which there will be uncertainties in government policies when there is a regime change. Since political instability can often result in turbulent conditions leading to several policy changes [68], we measured perceived policy instability with a categorical variable, ranging between 0 and 4 (0 = no obstacle, 1 = minor obstacle, 2 = moderate obstacle, 3 = major obstacle, 4 = very severe obstacle).

*Legal institutions*: According to Anderlini et al. [48], innovative firms’ output is affected by existing legal and economic institutions. WBES data provides report on whether firms perceive the legal system as a hindrance to their operations. The legal system variable is also a continuous variable, ranging between 0 and 4 (0 = no obstacle, 1 = minor obstacle, 2 = moderate obstacle, 3 = major obstacle, 4 = very severe obstacle).

### Control variables

*Firm size*: Size is anticipated to influence firms’ innovation activities. Following the literature (see, [69]), the paper controls for the impact of firm size on their ability to innovate. The WBES classifies firm size into small, medium, and large. Large firms are known to be innovative in comparison to small and medium sized firms because of economies of scale.

*Firm age*: According to Petruzzelli et al. [70], the age of a firm has a positive influence on its innovation performance. This paper measures firm age by the length of time firms are in operation since its year of establishment. We use the natural logarithm (Ln) to remove all potential biases.

*Managerial experience*: The innovation literature has well determined the nexus between years of top managers’ experience and its probable influence on firm-level innovations [60]. We expect that managerial experience could play key roles in overcoming the risks and negative effects of unfavourable institutional conditions [62]. Based on this, this research measured managerial experience using the question, "How many years of experience working in this sector does the top manager have?" We use the natural logarithm (Ln) to remove skewness and biases.

### Moderating variable

*Ownership structure*: The ownership structure of firms could affect how they can overcome the negative impact of institutional conditions. The ownership could also affect how firms may perceive the effects of unfavourable institutional conditions [53]. In this research, we measured ownership with the question, "What percentage of this firm do the largest owner or owners own?" We therefore introduced three moderating variables, with the first focusing on the percentage of firms that are *privately domestically owned*. The second focuses on the percentage of firms that are *privately owned by foreigners*. Lastly, *state-owned firms* denote the percentage of firms that are owned by the government. Table 1 below summarises the variables used in the empirical specification.

**Table 1:** Description of research variables.

Variable	Variable name	Description and measures
Dependent variable	Product innovation	Whether firms introduced new or significantly improved products or services (0, 1)
Covariates	Policy instability	Do firms perceive political instability as obstacle to their operations? (0–4)
	Legal system	Do firms perceive the courts as obstacle to their operations? (0–4)
	Informal competitions	Does firm compete against unregistered or informal firms? (0–1)
Control variables	Firm age	Log transformation of the total number of years of operations (years)
	Firm size	Total number of total employees (1–3)
	Managerial experience	Log transformation of the number of years of experience top manager has working in the sector.
Moderators	Private domestic ownership	Percentage of private domestic individuals, companies’ ownership (0–100).
	Private foreign ownership	Percentage of foreign domestic individuals, companies’ ownership (0–100)
	State ownership	Percentage of government or state ownership (0–100)

Source: Definitions adapted from the WBES

## Results and discussion

We begin the results discussion with the descriptive statistics of the variables shown in Table 2. The mean of product innovation is 0.325, indicating that about 33% of firms reported introducing significantly improved products or services during the last three years. Regarding the managerial experience variable, we noticed that the range was still high after standardization, implying that it greatly varies across the sampled firms. The same applies to the firm age variable; after standardization, the difference between the maximum and the minimum was large, indicating that age varies greatly in the sampled firms. The mean of the firm size variable is 1.563, indicating that the majority of the sample firms are small and microenterprises. As seen from the mean, the majority of the sampled firms are privately owned domestic firms (85.156), followed by foreign-owned firms (8.973). State-owned firms constitute the fewest firms in the sample with a mean of 0.694. The mean for competition from the informal sector is 0.577, denoting that approximately 58% of firm managers reported facing such competition. The mean for policy instability is 1.607, indicating that the majority of firms perceive policy instability as a minor obstacle. Finally, the mean for the legal system is 0.933, which means that firms perceive the legal system as no significant obstacle to their operations.

**Table 2:** Descriptive statistics and correlation coefficients.

	Mean	1	2	3	4	5	6	7	8	9	10
Product inno	0.325	1.000									
Firm size	1.563	0.018*	1.000								
Man. exp	2.710	-0.063*	0.150*	1.000							
Firm age	2.826	-0.019*	0.016*	0.086*	1.000						
Domestic	85.156	-0.095*	-0.161*	-0.038*	-0.005	1.000					
Foreign	8.973	0.085*	0.131*	0.007	0.013*	-0.751*	1.000				
State own	0.694	0.036*	0.091*	0.004	-0.000*	-0.207*	0.011	1.000			
Inform. com	0.577	0.144*	-0.114*	-0.016*	-0.016*	0.014*	-0.031*	-0.025*	1.000		
Policy inst.	1.607	-0.006	0.043*	0.084*	0.001	-0.040*	-0.003	0.004	0.070*	1.000	
Legal sys.	0.933	0.055*	0.033*	0.043*	0.020*	-0.033*	0.031*	0.014*	0.048*	0.391*	1.000

Source: Own calculations

Note:

* denotes correlation is statistically significant at the 0.05 significance level

As shown in Table 2, the correlation coefficients of the variables are shown to be very low, implying that collinearity or multicollinearity does not seem to be a major issue in the variables. We further used the variance inflation factor (VIF) to test for correlation; this helped us to avert possible deviations from multiple collinearities. The VIF results confirm that all variables have lower correlations with an overall mean of 1.27, demonstrating that there are no multicollinearity problems with the variables.

We first used the probit regression model to assess the various hypotheses. Tables 3 and 4 report the results of the regression of the three institutional condition variables and their interactions and product innovations. In Table 3, Model 1 is the baseline model with only control variables, and Model 2 adds the three main institutional variables. Model 3 includes the two-way interactions among the main institutional variables. Model 4 adds the three-way interaction of the main institutional variables. In Table 4, Model 1 includes the control variables. Model 2 includes the three institutional variables. Model 3 adds the three ownership structures. Model 4 adds the interaction of the three ownership variables with informal competition. Model 5 adds the interaction of the three ownership variables with perceived policy instability, while Model 6 adds the interaction of the three ownership variables with the legal system. To minimise the effects of heteroskedasticity in the variables used in the regression model, which could affect the coefficients’ significance, the robust standard errors estimator was employed in the multiple regression estimations [71].

**Table 3:** Probit results of institutional conditions on product innovation.

	(1)	(2)	(3)	(4)
Managerial experience	-0.041*** (0.004)	-0.041*** (0.004)	-0.119*** (0.012)	-0.119*** (0.012)
Firm size	0.015*** (0.004)	0.022*** (0.004)	0.061*** (0.013)	0.061*** (0.013)
Firm age	-0.009** (0.004)	-0.009* (0.004)	-0.026* (0.010)	-0.026* (0.010)
Informal competition [IC]		0.150*** (0.008)	0.423*** (0.032)	0.490*** (0.036)
Policy instability [PI]		-0.013*** (0.002)	-0.001 (0.012)	0.022 (0.013)
Legal system [LS]		0.024*** (0.003)	0.086*** (0.019)	0.156*** (0.025)
IC*PI			-0.029* (0.015)	-0.069*** (0.017)
IC*LS			0.052** (0.018)	-0.067* (0.033)
PI*LS			-0.021*** (0.006)	-0.052*** (0.009)
IC*PI*LS				0.051*** (0.012)
Country dummies	Yes	Yes	Yes	Yes
Sector dummies	Yes	Yes	Yes	Yes
_cons	-0.194*** (0.047)	-0.433*** (0.051)	-0.456*** (0.052)	-0.492*** (0.053)
*N*	21056	21056	21056	21056
pseudo *R*^2^	0.005	0.027	0.028	0.029

Robust standard errors in parentheses

* *p* < 0.05,

** *p* < 0.01,

*** *p* < 0.001

Note: results in Models 1 and 2 are marginal effects with Delta-method standard errors in parenthesis.

**Table 4:** Moderating role of ownership types on product innovation.

	(1)	(2)	(3)	(4)	(5)	(6)
Managerial experience	-0.041*** (0.004)	-0.041*** (0.004)	-0.119*** (0.012)	-0.120*** (0.012)	-0.117*** (0.012)	-0.121*** (0.012)
Firm size	0.015*** (0.004)	0.022*** (0.004)	0.038** (0.013)	0.045*** (0.012)	0.018 (0.012)	0.015 (0.013)
Firm age	-0.009** (0.004)	-0.009* (0.004)	-0.028** (0.010)	-0.026* (0.010)	-0.027** (0.010)	-0.029** (0.010)
Informal competition [IC]		0.150*** (0.008)	0.435*** (0.020)	0.339*** (0.079)		
Policy instability [PI]		-0.013*** (0.002)	-0.040*** (0.007)		0.067* (0.028)	
Legal system [LS]		0.024*** (0.003)	0.072*** (0.008)			0.027 (0.033)
Domestic owned [DO]			-0.002*** (0.000)	-0.003*** (0.001)	-0.001 (0.001)	-0.003*** (0.001)
Foreign owned [FO]			0.003*** (0.001)	0.003*** (0.001)	0.004*** (0.001)	0.003*** (0.001)
State owned [SO]			0.004** (0.002)	0.004 (0.002)	0.002 (0.002)	0.003 (0.002)
IC*DO				0.001 (0.001)		
IC*FO				-0.001 (0.001)		
IC*SO				0.003 (0.003)		
PI*DO					-0.001* (0.000)	
PI*FO					-0.001*** (0.000)	
PI*SO					0.001 (0.001)	
LS*DO						0.001*** (0.000)
LS*FO						-0.001*** (0.000)
LS*SO						-0.000 (0.001)
Country dummies	Yes	Yes	Yes	Yes	Yes	Yes
Sector dummies	Yes	Yes	Yes	Yes	Yes	Yes
_cons	-0.194*** (0.047)	-0.433*** (0.051)	-0.265*** (0.065)	-0.227** (0.079)	-0.118 (0.082)	0.0320 (0.070)
*N*	21056	21056	21056	21056	21056	21056
pseudo *R*^2^	0.005	0.027	0.035	0.031	0.013	0.016

Robust Standard errors in parentheses,

* p < 0.05,

** p < 0.01,

*** p < 0.001

Note: results in Models 1 and 2 are marginal effects with Delta-method standard errors in parenthesis.

Table 3 shows the probit regression results of institutional conditions on firms’ product innovation. The results in Model 2 show that any increase in informal competition and a favourable legal system leads to a marginal increase in product innovations. The increase ranges between 2 and 15 percentage points. As shown by the descriptive statistics in Table 2, firms in the sample do not consider these perceived institutional conditions to be major obstacles to their operations, including innovation activities. Contrary, any increase in perceived policy instability marginally reduced product innovation by 1 percentage point. The descriptive statistics results show that firms perceive policy instability as a minor obstacle, hence why it exerts a negative influence on product innovation. These results provide support for hypotheses 1a, 1b, and 1c. These firms are less affected by these external institutional conditions, such as informal competition and the legal system, probably because they are small businesses, as shown by the descriptive statistics results. Small businesses are usually flexible and agile compared to large firms, so they are more likely to adjust their strategies and activities rapidly to mitigate the impacts of these institutional conditions. They may also be inoffensive towards the competitors from the informal sector, which is why they might not be negatively affected by these unhealthy product market competitions. The objective levels of these variables vary across firms, implying that they could be perceived differently because firms may have different connections with the government or the legal system. The variability in these institutional conditions could also mean that firms have different levels of experience dealing with such conditions.

Firm size increases product innovation by 2 percentage points, according to firm characteristics. Firm age also has marginal effects on the likelihood of product innovations. To test Hypotheses 2, we introduced the interaction among the institutional variables. In Table 3, Model 3, when the interaction of the institutional variables is introduced into the regression equation, we find that the direction and significance of the main institutional coefficients remain unchanged. We find that the interaction between perceived policy instability and informal competition negatively impacts product innovations (-0.029), indicating that perceived policy instability negatively moderates this relationship. This could be explained by the fact that policy uncertainty raises the costs and risks of doing business, which is exacerbated when there is more unhealthy competition in the informal sector [3]. The interaction between perceived policy instability and the legal system also appears to negatively impact product innovations (-0.021). This result could be explained by the fact that firms are less likely to innovate when there is an increase in perceived policy instability, albeit the legal system could be effective, because increases in policy uncertainties can both inflate the cost of innovations or make firms abandon on-going innovation projects [72]. These results prove that our hypotheses 2a, 2b, and 2c are all supported. Furthermore, we find that the three-way interaction between perceived policy instability, informal competition, and the legal system positively impacts product innovation. This result means that even though perceived policy instability could have a potential deleterious impact on the relationship between informal competition and product innovation, a strong legal system can counterbalance its negative effects. An effective legal system will provide ample assurance that innovations will be protected, even when there are higher levels of policy uncertainty. This result provides support for Hypothesis 2d.

Again, when we put the various ownership structures into the equation and interact with these institutional variables, interesting results are seen, as shown in Table 4. We find that private domestic ownership positively moderates the relationship between the legal system and product innovations. This partly supports hypothesis 3. Since they are domestic-based firms, they may have the know-how to overcome the weaknesses of the legal system, and this could help them to innovate their new products. According to Blagojević and Damijan [73], private domestic firms are more likely to engage in state capture and informal payments; this can help them overcome an unfavourable legal environment. Private domestic ownership was also found to negatively moderate the relationships between perceived policy instability and product innovations. As shown in the descriptive statistics in Table 2, majority of these firms are domestic privately-owned, implying that they have a considerable portion of their operations and income connected to the domestic market. When perceived policy instability occurs, it can exacerbate uncertainty and unpredictability within the business environment. This increased uncertainty could discourage these domestic-owned firms from investing in R&D for new product development [74]. Also, private ownership could directly influence certain factors of product innovation such as technological advancements, access to resources, and the competitive landscape [75], but it may not directly moderate the relationship between informal competition and product innovation. This is because informal firms habitually operate in different areas which may not be directly affected by firms’ ownership structures. These results contradict our hypothesis 3.

Furthermore, we introduced foreign ownership into the equation and interacted it with the three institutional variables to test hypothesis 4. The results show that foreign ownership does not moderate the relationship between informal competition and product innovation. This means that hypothesis 4 is partially accepted but for informal competition. Though foreign firms are known to be innovative, when there are intense informal competitions, it can increase the cost of innovations over time. As the competition intensifies, it may discourage more innovation spending because it could lead to a situation where firms may not directly spend on innovation activities but protect themselves from these informal rivals. The results further show that the interaction between foreign ownership and the legal system has a negative impact on product innovations. Though the descriptive statistics results in Table 2 show that firms perceive the legal system to be favourable, our finding however indicates that it has a negative impact on foreign-owned firms as it weakens this relationship. The results further revealed that foreign ownership negatively moderates the relationship between perceived policy instability and product innovation. When policy uncertainties increase, foreign-owned firms may alter their investment priorities in response to policy changes. This perceived policy instability renders the business environment less attractive, hence foreign-owned firms may divert scarce resources away from innovation activities in favour of other regions with more stable and predictable conditions. These findings imply that our hypothesis 5 is partially supported for perceived policy instability and the legal system. Finally, we find full support for Hypothesis 5, that state ownership does not moderate the relationships with the three institutional variables and product innovations. This can be explained by the fact that their connections with the government provide them with protection from unfavourable institutional conditions. They are not likely to be negatively impacted by the legal system due to their affiliation with the government. State-owned firms will be able to influence governments to act against unfair informal competitors or ask for protection in times of policy instability. These results support the findings of Nguyen and Van Dijk [76] study in Vietnam, which concluded that state-owned firms are protected against unfavourable institutional conditions such as corruption.

### Robustness test

We carried out further analysis to test the robustness of the results described above. This paper further included other set of explanatory variables that may affect product innovation into the specification to reassess the results by testing the same hypotheses. Because this study is based on cross-sectional data, we account for potential endogeneity caused by cross-sectional issues and other heterogeneity [77]. According to Liu [78], survey data frequently contains variations in response patterns across different subgroups of respondents and that failing to account for this heterogeneity can lead to biassed estimates. Because our data is drawn from various industries and countries, there are compelling reasons to believe that unobserved heterogeneity and endogeneity play a role at the country and sector levels and that the effects of institutional conditions on innovation may differ as well. We attempt to mitigate the potential endogeneity problem by including firm, industry, and national-level controls [77, 78]. At the national level, we account for differences in the nation-states, though they are all African countries, by using the gross domestic product per capita. We also included country dummies as controls in the models. At the industry level, we included a sector dummy to show the sector in which the firm operates because competition intensity can vary across different sectors. At the firm-level, we also included a variable to capture regulatory quality measured by the amount of time firms’ senior managers spent meeting government regulations [79].

According to Lu and White [80], if the directions and significance of estimated regression coefficients do not vary much with the inclusion of different explanatory variables, it could be proof that they are robust and could be reliably deciphered to be true causal effects. The results are presented in Tables 5 and 6. The results show that the directions and magnitudes of the base-line regression coefficients remain unchanged. In the other models, when the interaction between the three institutional conditions and the various ownerships was introduced into the equation, the estimated regression coefficients and their various directions and significance remained the same. Therefore, we confirm that all our hypothesised relationships described above do not differ with the inclusion of other explanatory variables, hence buttressing the previous findings described above. As a consequence of our robustness check, which employs many other control variables, we conclude that our major findings remain unchanged.

**Table 5:** Robustness test for effects of institutional conditions.

	(1)	(2)	(3)	(4)
Managerial experience	-0.041*** (0.004)	-0.042*** (0.004)	-0.120*** (0.012)	-0.120*** (0.012)
Firm size	0.018*** (0.004)	0.023*** (0.004)	0.066*** (0.013)	0.066*** (0.013)
Firm age	-0.008* (0.004)	-0.009* (0.004)	-0.026* (0.010)	-0.026* (0.010)
Regulatory quality	0.001*** (0.000)	0.001*** (0.000)	0.004*** (0.000)	0.004*** (0.000)
GDP	0.034** (0.012)	0.032* (0.013)	0.086* (0.036)	0.088* (0.036)
Legal status	0.041*** (0.004)	0.036*** (0.004)	0.103*** (0.013)	0.103*** (0.013)
Informal competition [IC]		0.147*** (0.007)	0.404*** (0.033)	0.468*** (0.036)
Policy instability [PI]		-0.014*** (0.002)	-0.010 (0.012)	0.012 (0.013)
Legal system [LS]		0.024*** (0.003)	0.085*** (0.019)	0.151*** (0.025)
IC*PI			-0.021* (0.015)	-0.060*** (0.017)
IC*LS			0.051** (0.018)	-0.061 (0.033)
PI*LS			-0.021*** (0.006)	-0.050*** (0.009)
IC*PI*LS				0.049*** (0.012)
Country dummies	Yes	Yes	Yes	Yes
Sector dummies	Yes	Yes	Yes	Yes
_cons	-1.135*** (0.237)	-1.289*** (0.250)	-1.281*** (0.251)	-1.328*** (0.251)
*N*	21056	21056	21056	21056
pseudo *R*^2^	0.011	0.033	0.033	0.034

Standard errors in parentheses

* *p* < 0.05,

** *p* < 0.01,

*** *p* < 0.001

Note: results in Models 1 and 2 are marginal effects with Delta-method standard errors in parenthesis.

**Table 6:** Robustness test for moderating effects of ownership structures.

	(1)	(2)	(3)	(4)	(5)	(6)
Managerial experience	-0.041*** (0.004)	-0.042*** (0.004)	-0.121*** (0.012)	-0.122*** (0.012)	-0.119*** (0.012)	-0.124*** (0.012)
Firm size	0.018*** (0.004)	0.023*** (0.004)	0.045*** (0.013)	0.052*** (0.013)	0.026* (0.012)	0.024 (0.013)
Firm age	-0.008* (0.004)	-0.009* (0.004)	-0.028** (0.010)	-0.025* (0.010)	-0.026** (0.010)	-0.028** (0.010)
Regulatory quality	0.001*** (0.000)	0.001*** (0.000)	0.004*** (0.001)	0.004*** (0.000)	0.004*** (0.000)	0.004*** (0.000)
GDP	0.034** (0.012)	0.032* (0.013)	0.107** (0.037)	0.0825* (0.035)	0.114** (0.035)	0.113** (0.037)
Legal status	0.041*** (0.004)	0.036*** (0.004)	0.109*** (0.013)	0.106*** (0.013)	0.119*** (0.012)	0.120*** (0.013)
Informal competition [IC]		0.147*** (0.007)	0.428*** (0.020)	0.353*** (0.079)		
Policy instability [PI]		-0.014*** (0.002)	-0.045*** (0.007)		0.067* (0.028)	
Legal system [LS]		0.024*** (0.003)	0.072*** (0.009)			0.025 (0.034)
Domestic owned [DO]			-0.002*** (0.000)	-0.002*** (0.001)	-0.001 (0.001)	-0.003*** (0.001)
Foreign owned [FO]			0.003*** (0.001)	0.003*** (0.001)	0.005*** (0.001)	0.003*** (0.001)
State owned [SO]			0.005** (0.002)	0.004* (0.002)	0.002 (0.002)	0.004 (0.002)
IC*DO				0.001 (0.001)		
IC*FO				-0.001 (0.001)		
IC*SO				0.002 (0.003)		
PI*DO					-0.001** (0.000)	
PI*FO					-0.001*** (0.000)	
PI*SO					0.001 (0.001)	
LS*DO						0.001*** (0.000)
LS*FO						-0.000*** (0.000)
LS*SO						-0.000 (0.001)
Country dummies	Yes	Yes	Yes	Yes	Yes	Yes
Sector dummies	Yes	Yes	Yes	Yes	Yes	Yes
_cons	-1.135*** (0.237)	-1.289*** (0.250)	-1.258*** (0.258)	-1.067*** (0.249)	-1.196*** (0.251)	-1.034*** (0.260)
*N*	21056	21056	21056	21056	21056	21056
pseudo *R*^2^	0.011	0.033	0.041	0.037	0.019	0.022

Standard errors in parentheses,

* *p* < 0.05,

** *p* < 0.01,

*** *p* < 0.001

Note: results in Models 1 and 2 are marginal effects with Delta-method standard errors in parenthesis.

## Conclusion

This research provides a quantitative investigation of firms’ perceptions of institutional conditions and how they influence innovations in African economies. Specifically, we examined the influence of informal sector competitions, legal institutions, and perceived policy instability on product innovation. We also examined which firms can innovate in such unfavourable institutional conditions. We used pooled cross-sectional data from the World Bank enterprise survey for 42 African countries for our analysis. The pooled cross-sectional nature of the data means that there is greater variability of institutional conditions (intensity of competition, perceived policy instability, and a well-organized legal system) across firms at a particular time in a particular country. Our empirical estimation results point to the following main findings: about 33% of the sample firms reported having innovated their new products. The results demonstrated that institutional conditions such as informal competitions and the legal system, positively and marginally influence product innovations, while perceived policy instability marginally decreases it. We also discovered that managerial experience and firm age marginally decrease product innovation. Another key finding is that perceived policy instability negatively moderates the relationships between the legal system and firms’ product innovation but positively moderates the relationship between informal competitions, legal system, and product innovation. The study also found that the legal system positively moderates the relationship between informal competition and product innovation. However, the combinations of these three institutional conditions positively impact product innovation. Private domestic as well as foreign ownerships negatively moderate the relationship between perceived policy instability and product innovation, implying that it diminishes domestic and foreign firms’ innovations. Contrary to popular belief, foreign-owned firms are less likely to innovate their products when the legal system is unfavourable. Finally, the results have shown that state ownership does not moderate the relationships between the institutional conditions outlined in this paper. These institutional conditions do not significantly affect SOEs abilities to innovate their product.

These results offer significant contributions to the growing institutional theory literature. First, our results have proven that perceived competition from the informal sector and legal institutions positively influence firms’ product innovations. The results also proved that the combination of informal sector competition, perceived policy instability and legal institutions positively impact product innovation. These results show that formal institutional conditions provide the requisite structural environment for innovations to thrive in emerging markets, which are usually characterised as having weaker institutions. Contrary to our expectations, firms embedded in such institutional environment, are more likely to be positively impacted by their joint effect in terms of new product development. These results contribute to the recent growing literature on institutional theory from the perspective of developing countries [3, 4, 81]. Our research builds upon the work of Dunyo and Odei’s [40] study on Thailand, which found that country-specific institutional conditions negatively influence technological and non-technological innovation outcomes. Our results provide useful insight that we need greater variability in institutional conditions to overcome potential endogeneity issues that could arise when dealing with cross-sectional data. Our results have shown the importance of specific features of institutional conditions based on diverse firms from different African economies. The results are robust and consistent with the inclusion of different explanatory variables.

Furthermore, our study solely focused on measuring innovations from the perspective of new product development. The empirical results provide new insight into understanding the role institutional conditions play in the firms’ product innovations. Our sole measure means that we provide a detailed understanding of how firms perceive institutional conditions and their ability to influence product innovation. We have also shown that the combination of perceived policy instability and an unfavourable legal environment negatively influence product innovations. Under these current institutional environment, foreign-owned firms are less likely to innovate their products. These institutional conditions are very important as they could negatively impact new product development; hence, they deserve various central governments attention. The inclusion of the various ownership structures into the analysis also helped us determine and understand which firms could be affected by these unfavourable conditions and how they can innovate amidst such conditions. The result showed that foreign-owned firms are not able to innovate in an unfavourable legal environment, because it reduces their incentives to innovate their new products. These findings contribute to the literature on institutional conditions and how they could be influenced by various firms’ ownership structures.

Our findings have important practical implications for policymakers and practitioners across African countries. The results have demonstrated that perceived institutional conditions such as informal sector competitions, and the legal system positively influence new product development. African policy makers therefore need to be fully aware that these perceived prevailing institutional conditions influence firms’ product innovation outcomes. These results could be of importance to policy makers and practitioners as they can be helpful in identifying the country-specific institutional conditions that could be ameliorated to spur firm-level innovations. Our results have also shown that the perceived policy instability negatively impacts the relationships between informal competition, the legal system, and firms’ new product development. This is a call to African policymakers to consider making the legal system more corporate-friendly. Issues such as the exorbitant cost of starting or defending litigation, under-funded judicial institutions, and the abnormal delays in justice delivery caused by recurrent adjournments require stakeholders’ attention. In terms of overcoming the harmful effects of perceived policy instability which is rampant in the West African region, this study recommends policy makers and governments taking over administration to ensure policy continuity when there are changes in government. Policy makers also need to examine the impacts of policy changes on both domestic and foreign-owned firms and develop specific targeted strategies that promote policy stability, predictability as well as their effective implementation. This could help reduce their potential negative impact on these firms’ abilities to innovate. Our findings indicate that foreign ownership has a negative impact on the relationship between the legal system and product innovation. The results show that the legal environment in these emerging markets is favourable for domestic owned firms but not foreign owned firms. This calls for policymakers and foreign owners’ continuous collaboration to assess aspects of the legal system that are unfavourable to their operations since they are proven to be vital in knowledge and technology transfers to developing countries [20, 34]. These regular engagements could help understand foreign companies concerns, challenges, aspects of the legal system unfavourable to their operations, and various suggestions on how to improve the legal environment.

As expected, this research acknowledges a few limitations that could be improved in future research. First, this study only considered product innovation, implying that the results should not be interpreted to include other measures not considered. Second, combining all African countries as a single analytical unit could possibly have affected our results. African countries are dynamic with fast-changing institutional conditions; this implies that changes in the prevailing institutional conditions are imminent. Our data covered most African economies but not all countries; hence, the results should not be generalised. Heterogeneity is also a key concern that should be considered as African countries differ in terms of institutions, socio-economic development, and historical development, despite being in the same geographic region. This research was based on pooled cross-sectional datasets, to thoroughly capture the dynamics and long-run effects of perceived institutional conditions on innovations in African countries, we recommend future research consider using longer panel datasets when they become available. Finally, our results need to be interpreted with caution as the data is from a perception survey, reflecting firm managers’ perceptions of prevailing institutional conditions. They may not reflect the true state of institutional conditions as perceptions could be subjected to respondent biases and misinformation which may not always align with objective realities. We therefore recommend future studies to consider the balanced approach that blends both objective measures as well perception-based indicators to deepen the understanding of how institutional conditions impact firms’ innovations in African countries.
